# Spatial temperature distribution in human hairy and glabrous skin after infrared CO_2 _laser radiation

**DOI:** 10.1186/1475-925X-9-69

**Published:** 2010-11-08

**Authors:** Ken S Frahm, Ole K Andersen, Lars Arendt-Nielsen, Carsten D Mørch

**Affiliations:** 1 Center for Sensory-Motor Interaction (SMI), Aalborg University, Fredrik Bajers vej 7D-3, DK 9220 Aalborg, Denmark

## Abstract

**Background:**

CO_2 _lasers have been used for several decades as an experimental non-touching pain stimulator. The laser energy is absorbed by the water content in the most superficial layers of the skin. The deeper located nociceptors are activated by passive conduction of heat from superficial to deeper skin layers.

**Methods:**

In the current study, a 2D axial finite element model was developed and validated to describe the spatial temperature distribution in the skin after infrared CO_2 _laser stimulation. The geometry of the model was based on high resolution ultrasound scans. The simulations were compared to the subjective pain intensity ratings from 16 subjects and to the surface skin temperature distributions measured by an infrared camera.

**Results:**

The stimulations were sensed significantly slower and less intense in glabrous skin than they were in hairy skin (MANOVA, p < 0.001). The model simulations of superficial temperature correlated with the measured skin surface temperature (r > 0.90, *p *< 0.001). Of the 16 subjects tested; eight subjects reported pricking pain in the hairy skin following a stimulus of 0.6 J/cm^2 ^(5 W, 0.12 s, d1/e^2 ^= 11.4 mm) only two reported pain to glabrous skin stimulation using the same stimulus intensity. The temperature at the epidermal-dermal junction (depth 50 μm in hairy and depth 133 μm in glabrous skin) was estimated to 46°C for hairy skin stimulation and 39°C for glabrous skin stimulation.

**Conclusions:**

As compared to previous one dimensional heat distribution models, the current two dimensional model provides new possibilities for detailed studies regarding CO_2 _laser stimulation intensity, temperature levels and nociceptor activation.

## Background

Lasers have previously been used in several pain research applications [1-3]. Lasers have the ability to transiently deliver large amounts of energy to the skin in a highly reproducible way without concurrent mechanical stimulation. Different lasers (infrared and visible) are therefore very useful for quantitative sensory testing and recording of time locked evoked potentials [1]. When using lasers for pain stimulation it is imperative that the skin is not damaged due to burns, hence, it is essential to use intensities above the nociceptive threshold but below the tissue damage threshold [1].

The cutaneous penetration depth of the emitted laser energy depends on the wavelength of the photons [4]. Infrared CO_2 _lasers (10.6 μm) have a very short penetration depth (20 μm) as the energy has to be absorbed by the water content in the stratum corneum and other layers of the epidermis [2,5]. Tillman et al. [6] investigated the depth of C-nociceptive fibers in monkeys and found the average to be 201 μm in hairy skin [6]. For the hairy skin on the back of humans, Hilliges et al. [7] found that Aδ fibers penetrated from dermis into the epidermis. In the glabrous skin the epidermis is generally less innervated by nociceptors than in hairy skin [7]. Atherton et al. [8] found intraepidermal fibers which showed antibody immunostaining to the heat sensitive TRPV1, indicating that heat sensitive fibers are present in the epidermis. However, no evidence was found that the fibers extended into the stratum corneum [8]. Since approximately 90% of the infrared CO_2 _laser energy is absorbed in the very superficial stratum corneum and epidermis it has to be passively conducted deeper into the skin to activate the nociceptors. Therefore, part of the delay in the transduction process leading to activation of the nociceptors must be due to passive conductance of the thermal energy through the superficial skin layers.

Previous studies have investigated the CO_2 _laser evoked responses (latencies and pain intensities) from 2 different Aδ fiber populations (type I and type II) of glabrous versus hairy skin [3,9]. Type I has a response threshold of 53°C and a response latency of 10 ± 12 s whereas type II has a response threshold of 46°C and a response latency of 0.12 ± 0.08 s [3]. In hairy skin both types are present, whereas, in glabrous skin only type I is present. According to Treede et al. [3] this explains why pricking pain cannot be elicited in glabrous skin. In contrast only one type of C fibers exists (ignoring different populations of TRP channels in C fibers), and Treede et al. [3] found that C fibers has an activation threshold of 41°C and a response latency of 0.1 ± 0.17 s. These C fibers have different transduction latencies in glabrous than they have in hairy skin (0.1 s in hairy vs. 0.25 s in glabrous skin) [3]. This difference might be related to different thermal properties, moreover, due to thicker epidermis in the glabrous skin. The epidermis has lower thermal conductivity than the dermis and, therefore, isolates the thermal energy from the nerve endings. Hence, in the present study it was hypothesized that two identical stimuli will be perceived less painful in glabrous skin due to the thicker epidermis.

Mathematical modeling can predict the temperature increase evoked by a CO_2 _laser stimulus at different depths. Simple 1D heat transfer models have been used [2,5,10]. However, those models cannot incorporate the different thermal properties of the different skin layers e.g. stratum corneum, vital epidermis and dermis [2,5]. Furthermore, 1D models only predict the heating directly below the center of the laser beam. Theoretically, a small beam width (~1 mm) might not activate any nociceptors. Furthermore, a 1D model assumes that all heat energy is transported downwards into the skin, not radially spreading and in addition the laser beam profile cannot be taken into account.

2D axial and 3D models exist [11-13], but these models are not developed for the purpose of pain research. Haimi-Cohen et al. [14] developed a somewhat rigid model to investigate nociceptor activation following CO_2 _laser stimulation; however, the model did not incorporate different thermal properties of different skin layers. Nor were the modeled results experimentally validated [14]. Nevertheless, significant differences in the temperature profiles at the skin surface and receptor level were observed [14]. Based on the previously developed methods [11-14] a new, more flexible model will be developed. The model will incorporate the thermal properties of different skin layers, apply finite element modeling to limit the amount of analytical assumptions and will be validated through experimental trials.

The aims of this study were 1) to develop a 2D axial model of the temperature distribution at different depths in the skin after infrared CO_2 _laser stimulation, 2) to validate the model by measuring surface temperatures using thermography, and 3) relate the thermal parameters to the pain intensity reports.

## Methods

### Subjects

Sixteen healthy subjects (age range 23-34 years, 9 females, 7 males) participated in this study. Informed written consent was obtained from all subjects in accordance with the Helsinki declaration and approval for the study was obtained from the local ethics committee (N20080026).

**Figure 1:**
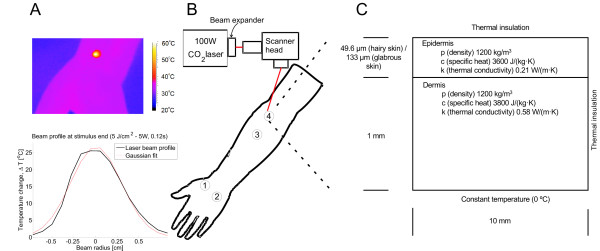
**The figure illustrates the experimental setup and the parameters used for the finite element model**. In A the laser beams spatial profile is seen in the infrared camera. The slight skewed look of the profile is most likely due to fact that the cameras line of sight was not perpendicular to the skin surface. But it could also be due to the poor spatial resolution of the infrared camera and an inaccuracy of the laser optics. However, a Gaussian fit of the laser beam showed that the laser beam fits a Gaussian profile with an r^2 ^of 0.984. In B the experimental setup is displayed. It consists of a 100 W CO_2 _laser with a beam expander and scanner head. The latter directs the laser beam to the desired skin sites. In C the principle behind the finite element model is displayed including thermal constants, geometric sizes and simulation properties. The lower boundary is kept at a constant temperature (0°C). Note that the initial temperature in the model is 0°C. This mimics how the thermal energy deeper into the tissue which is the primary cooling effect during laser stimulation.

During the experiments the subjects were seated in a chair with the dorsum of their left hand and forearm placed flat on a table with the volar forearm perpendicular to the laser beam (Figure 1). The subject and investigator wore protective goggles for the duration of the experiment.

### Epidermal thickness measurement

The epidermal thickness of each subject was measured at two sites on the left palm (root of thumb (site 1) and root of lateral palm (site 2)) and two skin sites on the left volar foream, (5 cm (site 3) and 10 cm (site 4) from the elbow) (Figure 1) using a Derma scan, high resolution, ultrasound scanner (50 MHz)(Cortex Technologies A/S, Denmark). The sites in the forearm were shaved prior to the assessment. The distances from the test sites to the spinal cord were measured.

### Laser stimulation, infrared recording, and subjective pain rating

Heat stimuli were produced by a Synrad Firestar 100 W CO_2 _(10.6 μm wavelength, beam diameter 2.2 ± 0.2 mm (d1/e^2^) equipped with a 5× beam expander, beam area 1.02 cm^2^) laser controlled by a Synrad UC-1000 control box, and a custom made Labview (National Instruments, United States) application. The laser power was calibrated before each experiment, using a Coherent PM150/FieldMax II power meter. Two output power levels were used, 1 W and 5 W. Five stimulation energies were applied by varying stimulation duration and intensity; 0.2 J/cm^2 ^(1 W, 0.2 s, d1/e^2 ^= 11.4 mm), 0.4 J/cm^2 ^(1 W, 0.4 s, d1/e^2 ^= 11.4 mm), 0.6 J/cm^2 ^(1 W, 0.6 s, d1/e^2 ^= 11.4 mm), 0.8 J/cm^2 ^(1 W, 0.8 s, d1/e^2 ^= 11.4 mm), and 0.6 J/cm^2 ^(5 W, 0.12 s, d1/e^2 ^= 11.4 mm)(Table 1). An inter-stimulus interval of at least one minute was used to ensure sufficient cooling of the skin between stimuli.

**Table 1:** The table displays the experimental protocol used.

Repetitions at each site	Hairy skin		Glabrous skin	
Site number	Site 1	Site 2	Site 3	Site 4

0.2 J/cm^2 ^(1 W, 0.2 s, d1/e^2 ^= 11.4 mm)	2	2	2	2

0.4 J/cm^2 ^(1 W, 0.4 s, d1/e^2 ^= 11.4 mm)	2	2	2	2

0.6 J/cm^2 ^(1 W, 0.6 s, d1/e^2 ^= 11.4 mm)	2	2	2	2

0.8 J/cm^2 ^(1 W, 0.8 s, d1/e^2 ^= 11.4 mm)	2	2	2	2

0.6 J/cm^2 ^(5 W, 0.12 s, d1/e^2 ^= 11.4 mm)	4	4	4	4

Each 1 W stimulation was delivered twice at each site. This added up to a grand total of 32 stimulations. Each 5 W stimulation was delivered 4 times at each site corresponding to a grand total of 16 stimulations.

An infrared camera (Thermovision 900, Agema Infrared system, Sweden) continuously monitored the skin temperature (30 Hz frame rate), spatial resolution 7-10 pixels pr. cm. A 1 × 1 cm aluminum square was placed on the skin adjacent to the stimulation site (~5 cm from the stimulation site) for spatial calibration in the infrared recordings.

The volunteers were instructed to press a button as soon as any sensation was felt, to give an indication of the perception latency caused by the transduction process in the skin and sensory system. The perception latencies were defined as the time from stimulus onset until the button was pressed. Furthermore, the subjects were instructed to rate the pain intensity from the laser stimulation on a continuous Visual Analog Scale (VAS) anchored by '0' as no sensation, '5' as pain threshold, and '10' as maximum pain. The scale was presented verbally and visually to the subjects; the scale was marked with 'No sensation' at 0, 'Pain threshold' at 5 and 'Maximum pain' at 10.

### Experimental protocol

A total of four stimulation sites were used; two in hairy skin on the left volar forearm; and two in the glabrous skin on the left palm (Figure 1). The order of stimulation sites was randomized in a balanced way so no site was ever stimulated with two consecutive stimulations. A single stimulation paradigm included all four stimulation sites. Each of the 1 W paradigms were tested 8 times resulting in a grand total of 32 stimulations. Each of the 5 W paradigms was tested 4 times, meaning a grand total of 16 stimulations (Table 1). The 1 W power setting was mainly chosen for model validation, knowing that some of these stimuli would not evoke any sensations in the subjects.

### Finite element modeling

The model for the temperature profile was based on the bioheat equation [15]. By ignoring any temperature change due to perfusion which is legitimate due to the sparse perfusion of the upper dermis and epidermis [4], the bioheat equation can be reduced to (Eq. 1).

(1) $$pc\frac{\partial T(\text{r},t)}{\partial t}-\nabla (k\nabla T(\text{r},t))=Q(\text{r},t)$$

*T *is the tissue temperature, *t *is time, *ρ *is the tissue density, *c *is the specific heat of the tissue, *k *is thermal conductivity, ***r ***is the spatial coordinate, and *Q *is the heat source [4].

The simplified model (Eq. 1) was solved numerically by finite element modeling (FEM; COMSOL multiphysics, Sweden). The FEM was created using a 2D axial symmetrical geometry. The model was comprised of two vertical rectangles placed on top of each other each representing epidermis and dermis respectively (Figure 1). The mesh in the model was auto generated in COMSOL by setting the free mesh parameter to 'Normal', this generated 1417 mesh elements in the hairy skin model and 435 mesh elements in the glabrous skin model. The temperature change (∆*T*) was normalized by setting the initial temperature change to 0°C, ignoring any phase changes. The temperature of the lower boundary was kept constant at the initial temperature (∆*T *= 0°C). The upper boundary (the skin surface) was defined as thermally insulated, ignoring any heat transfer into the adjacent air. The spatial beam profile of the laser was modeled as Gaussian with a standard deviation of 2.85 mm (based on model fitting of the laser system, which corresponds to a 1/e^2 ^diameter of 11.4 mm). The light absorption in the tissue was modeled as an exponential decay using Beer-Lamberts law. Combining these conditions, *Q *was modeled as outlined in Eq. 2. The thermal constants and geometry used in the model are found in Figure 1.

(2) $$Q(\text{r},t)=\text{​}P_{in}\mu_{a}e^{\left(-\mu_{a}z\right)}\frac{1}{\sigma \sqrt{2\pi }}e^{\left(\frac{-r^{2}}{2\sigma^{2}}\right)}$$

*P*_*in *_is the laser power setting, *μ*_*a *_is the absorption coefficient of the tissue (50000 m^-1^, based on [5] and average water content of 60% in stratum corneum and the rest of epidermis), *z *is the depth from the tissue surface, *σ *is standard deviation of the Gaussian beam profile equal to 2.85 mm for the present laser system (Synrad Firestar 100 W CO_2 _laser, equipped with 5× beam expander) and *r *is the distance to center of the beam.

### Data analysis and statistics

Spatial surface temperature profiles at the center of the stimulation sites were obtained from the thermographic images starting 1 s before laser onset and lasting for 11 s. The temperature profiles were normalized to the surface skin temperature at the stimulation site 1 s before laser onset. Mean surface temperature profiles were calculated for each stimulation paradigm. The results were compared to the FEM output by correlating the modeled and measured maximum temperature profiles.

The temporal temperature profile at the center of the stimulation at different depths (0 μm, 100 μm, 200 μm, 300 μm, 400 μm, 500 μm and the epidermal ridge - 50 μm for hairy and 133 μm for glabrous skin) were extracted from the FEM.

The correlation coefficient (r^2^) between the reported VAS score and modeled maximum temperature at the center of the beam at several depths (1 μm step) were calculated to estimate the skin depth of the heat sensitive nociceptors in different skin types.

Using two separate 2-way analysis of variance, the differences between the VAS and latencies following all 1 W stimulations were examined for both skin types. The factors were skin type and stimulation energy. A Tukey post-hoc test was applied in the case of any significant difference. A Student's t-test was used to assess the differences in the VAS following the 5 W stimulations. A non-paired t-test was used to compare the perception latencies following the 5 W stimulations (not all stimulations evoked sensation in the subjects, creating unequal latency samples). In the statistical tests of the 1 W data, the lowest stimulation energy, 0.2 J, was left out, since it did not evoke any sensation in most subjects.

Two Multivariate Analysis of Variances (MANOVAs) were used to compare the modeled subsurface temperatures; the magnitude of the maximum modeled temperature and the delay to maximum temperature after stimulus end. A Tukey post-hoc test was applied in case of significant differences including tests for 2-way interaction in the 1 W data. In the 1 W data no 3-way interaction was tested since the modeling data only provided a single output for each combination of all three factors. In the 5 W data no interaction was tested since the modeling data only provided one combination of the factors.

All results are presented as mean ± one standard deviation (SD).

## Results

### High resolution ultra sound scans

The geometry of the FEM was determined using high resolution ultrasound scans. The grand averages for all 4 sites in all 16 subjects for the epidermal thickness were in the glabrous skin found to be 0.101 ± 0.04 mm for site 1 and 0.164 ± 0.07 mm for site 2. In the hairy skin it was found to be 0.049 ± 0.01 mm for site 3 and 0.050 ± 0.02 mm for site 4. For use in the model, the glabrous skin grand averaged epidermal thickness was therefore 0.133 ± 0.06 mm; while the hairy skin grand averaged epidermal thickness was 0.050 ± 0.01 mm. The dermal thickness was measured to be 1 mm in both skin types.

### Pain intensity and perception latencies reported

The reported sensations and latencies can be seen in Table 2.

**Table 2:** The table displays the mean sensations and perception latencies reported.

	Skin type	**Glabrous skin **(site 1 & 2)				**Hairy skin **(site 3 & 4)			
	**Duration **(s)	0.2	0.4	0.6	0.8	0.2	0.4	0.6	0.8

**1 W**	**VAS **(mean ± SD)	0.40 ± 0.48	0.43 ± 0.67	0.66 ± 0.92	1.15 ± 1.31	0.48 ± 0.68	1.23 ± 1.34	2.01 ± 1.58	2.97 ± 1.86

	**Perception latency **(s) (mean ± SD)	1.98 ± 0.88	1.61 ± 0.50	1.56 ± 0.92	1.51 ± 0.47	1.69 ± 1.13	1.30 ± 0.37	1.21 ± 0.20	1.15 ± 0.32

	**Duration **(s)	0.12				0.12			

**5 W**	**VAS **(mean ± SD)	1.29 ± 1.73				4.82 ± 1.89			

	**Perception latency **(s) (mean ± SD)	1.34 ± 0.48				0.55 ± 0.26			

The pain intensities were significantly higher in hairy skin than in glabrous skin and the VAS scores increased as stimulus energy increased when 1 W was applied (repeated 2-way ANOVA and Tukey post-hoc test, *p *< 0.001). The perception latencies were longer when 1 W stimulations were applied to glabrous skin than when applied to hairy skin (repeated 2-way ANOVA, *p *< 0.001). No differences were found between stimulation energies (repeated 2-way ANOVA, *p *= 0.3).

The pain intensities were higher when 5 W stimulations were applied to hairy skin than when applied to glabrous skin (Student's t-test, *p *< 0.001). The perception latencies were longer when 5 W stimulations were applied to glabrous skin than when applied to hairy skin (t-test, *p *< 0.001).

For the highest stimulus intensities the subjects reported warm burning sensations in the glabrous skin, but in hairy skin they reported a pinprick pain.

### Maximum surface temperature

**Figure 2:**
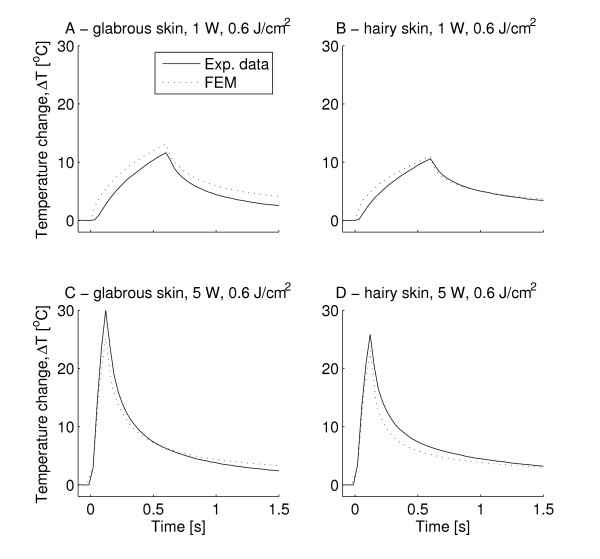
**The figure illustrates the maximum surface temperature during laser heating and cooling**. Time, t = 0 is set at stimulus onset. The figure depicts the mean experimental data compared to the modeled based on a finite element model (FEM). The experimental data are depicted as mean values (Exp. data) of the experimental data. A) glabrous skin, 1 W, 0.6 s, 0.6 J/cm^2 ^B) hairy skin, 1 W, 0.6 s, 0.6 J/cm^2 ^C) glabrous skin, 5 W, 0.12 s, 0.6 J/cm^2 ^D) hairy skin, 5 W, 0.12 s 0.6 J/cm^2^. The increase in surface temperature was larger in glabrous skin than in hairy skin. The maximum temperature change reached was approximately doubled when using a 5 W power setting instead of 1 W (A & B vs. C & D).

Typical data of the temporal profile of the maximum temperature is depicted in Figure 2.

There was strong correlation between the modeled temporal profiles and the experimental data, r > 0.95 (except for the lowest energy tested (0.2 J), r = 0.90), for all cases the correlation was significant (*p *< 0.001).

**Figure 3:**
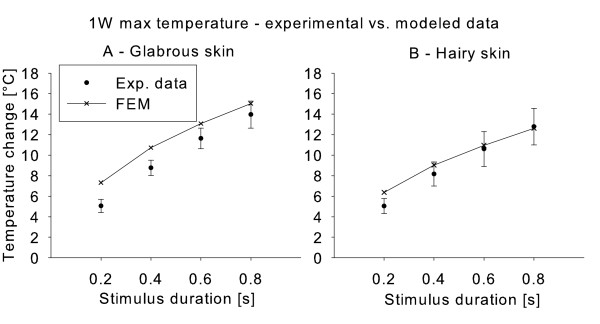
**The figure illustrates the experimental (Exp. data) and modeled maximum skin temperatures (FEM) at stimulus end for the 1 W stimulations in both glabrous and hairy skin for all four tested stimulation durations (0.2, 0.4, 0.6, and 0.8 s)**. 95% confidence intervals are marked on the experimental data.

Furthermore, in the hairy skin the modeled maximum skin temperature fell within the 95% confidence intervals of the experimental data for all but one stimulation durations applied at 1 W (Figure 3-B); while in the glabrous skin only one did (Figure 3-A).

### Spatial temperature profiles

**Figure 4:**
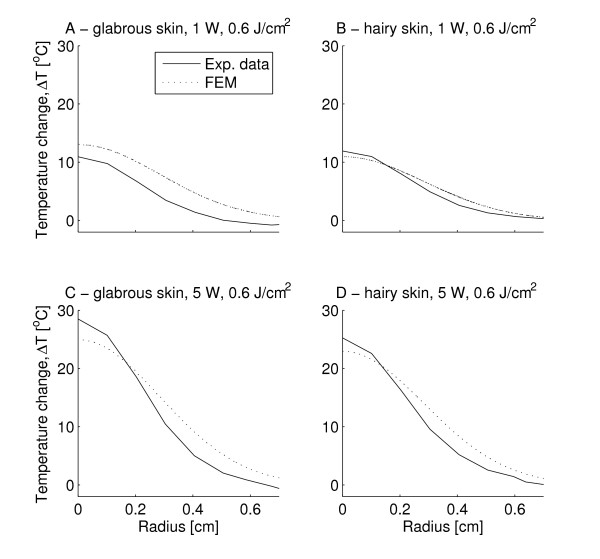
**The figure illustrates the spatial surface temperature profile at stimulus end (maximum surface temperature) for both mean experimental data (Exp. data) and modeled temperatures based on a finite element model (FEM)**. A) glabrous skin, 1 W, 0.6 s, 0.6 J/cm^2 ^B) hairy skin, 1 W, 0.6 s, 0.6 J/cm^2 ^C) glabrous skin, 5 W, 0.12 s, 0.6 J/cm^2 ^D) hairy skin, 5 W, 0.12 s, 0.6 J/cm^2^. In all plots, it is seen that the temperature change 0.6 cm from the beam center at stimulus termination was less than 5°C. Even though the maximum temperature (at the beam center, 0 cm) for the 5 W power setting was approximately the double of the 1 W power setting, the measured and simulated temperature changes more than 0.5 cm from the beam center was identical.

The spatial temperature profiles of the model were similar to the infrared recordings (Figure 4). The spatial profiles for the three other 1 W stimulus energies were also found close to the infrared recordings (not displayed). The maximum temperature reached at the end of the stimulus (at the beam center, 0 cm) was approximately doubled when using the 5 W (0.12 s, 0.6 J/cm^2^) stimulation compared to the 1 W (0.6 s, 0.6 J/cm^2^) stimulation (Figure 4). However, the evoked temperature change more than 5 mm from the beam center was comparable (Figure 4).

### Sub-surface temperature profiles

**Figure 5:**
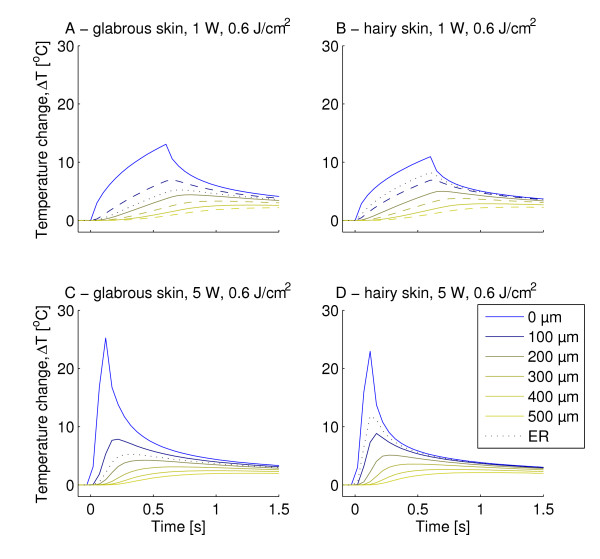
**The figure illustrates the surface and subsurface temporal maximum temperature profiles calculated by the finite element model during laser heating and subsequent cooling (pulse duration listed)**. The surface temperature profile and six different depths are displayed (100, 200, 300, 400, 500 μm and the depth of the epidermal ridge (ER), which is 130 μm in glabrous skin and 50 μm in hairy skin). It is seen that the delay to reach the maximum temperature after stimulus termination increased with depth, as the heat energy took longer time to diffuse into the skin. Comparing the temperature profile at the ER in hairy and glabrous skin, it is seen that following 5 W stimulations the maximum temperature was almost twice as high in hairy skin as it was in glabrous skin. For the 1 W data the differences were not as pronounced, because the slower heating allows better time for the heat time to diffuse into the skin. A) glabrous skin, 1 W, 0.6 s, 0.6 J/cm^2 ^B) hairy skin, 1 W, 0.6 s, 0.6 J/cm^2 ^C) glabrous skin, 5 W, 0.12 s, 0.6 J/cm^2 ^D) hairy skin, 5 W, 0.12 s, 0.6 J/cm^2^.

**Figure 6:**
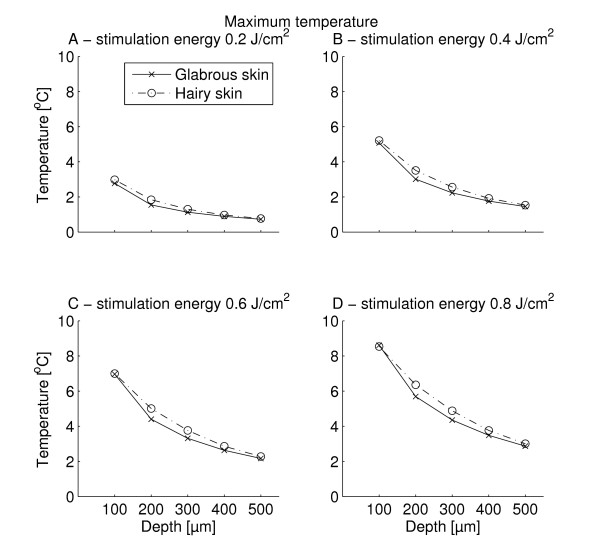
**The figure depicts the maximum temperature reached at different depths following 1 W stimulations for all four stimulation energies tested (0.2, 0.4, 0.6, and 0.8 J/cm**^**2**^**) at five different depths (100, 200, 300, 400, and 500 μm)**. It is seen that the maximum temperature reached increased as stimulation energy increased (*p *< 0.001). Furthermore, it is seen that the maximum temperature decreased with depth (*p *< 0.001). For similar depths, all, but one stimuli created temperatures in hairy skin which were slightly higher than the corresponding stimuli did in glabrous skin (3-way MANOVA, *p *< 0.001).

**Figure 7:**
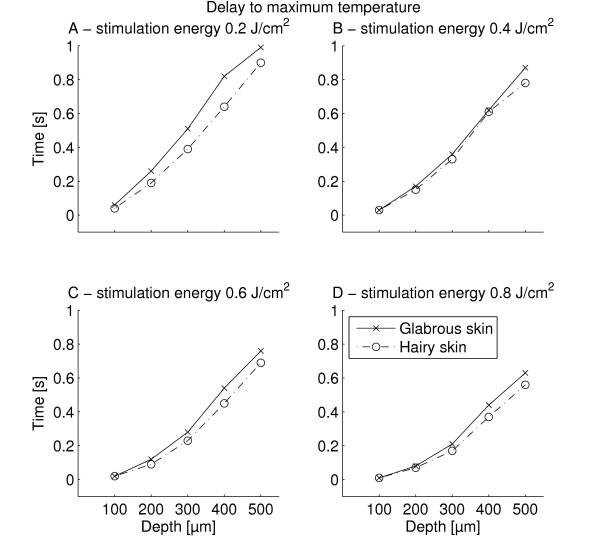
**This figure depicts the delay (after stimulus end) to reach maximum temperature following 1 W stimulations for all four stimulation energies tested (0.2, 0.4, 0.6, and 0.8 J/cm**^**2**^**) at five different depths (100, 200, 300, 400, and 500 μm)**. The delay to reach the maximum temperature decreased as stimulation energy increased (*p *< 0.001). Furthermore, the delay generally increased with depth (*p *< 0.001). The delay in glabrous skin was generally higher than in hairy skin (3-way MANOVA, *p *< 0.001).

**Figure 8:**
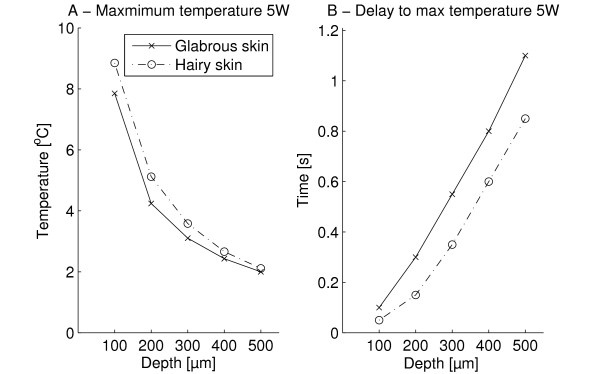
**The figure illustrates the modeled sub-surface temperatures following 5 W stimulations (5 W, 0.12 s, 0.6 J/cm**^**2**^**) in both glabrous and hairy skin for five different depths (100, 200, 300, 400, and 500 μm)**. Furthermore, the delay to reach maximum temperature after stimulus end is displayed. The maximum temperature reached decreased with depth (*p *< 0.001). The delay before reaching the maximum temperature increased with depth (*p *< 0.001). The sub-surface temperature was generally higher in hairy skin than in glabrous skin. Conversely the delay was larger in the glabrous skin than in hairy skin (2-way MANOVA, depth: *p *< 0.001, skin type: *p *= 0.001).

The temperature development at the surface and at six different depths was extracted from the FEM (Figure 5). It is evident, that sub-surface temperatures were shifted both in time and magnitude meaning that lower maximum temperatures were observed for increasing skin depth and the delay of the peak temperature also increased with skin depth. The maximum sub-surface temperatures reached for all 1 W stimulations are depicted in Figure 6. The delays after stimulus end to reach maximum subsurface temperature for all 1 W stimulations are depicted in Figure 7. For all but one stimulation the subsurface temperatures in hairy skin were generally higher than in glabrous skin (Figures 6 &8). The delay after stimulus end to reach maximum temperature in glabrous skin was for all but two stimulations higher than the delay in hairy skin (Figure 7). Both the maximum temperature reached and the delay to the peak temperature showed a significant differences between skin type, skin depth and stimulation duration (3-way MANOVA, *p *< 0.001). For both dependent variables (maximum temperature reached and delay to peak temperature) a 2-way interaction was seen between all factors. A Tukeys post-hoc test showed significant differences both between all 1 W stimulation durations, and between all tested depths. Furthermore, the post-hoc test showed that between hairy and glabrous skin there were no interaction for 100 μm and 500 μm, but for all other depths.

The maximum sub-surface temperature and delay after stimulus termination for 5 W stimulations are depicted in Figure 8 with results resembling the 1 W stimulation. The delay to reach maximum temperature increases with depth and the maximum temperature reached decreases with skin depth. The sub-surface maximum temperature reached was generally higher in hairy skin, while the delay was higher in the glabrous skin. Significant differences between skin type and skin depth were found (2-way MANOVA, *p *< 0.001). The maximum temperatures at 100 and 200 μm were significantly higher than at all other skin depths (Tukey post-hoc, *p *< 0.001). For 300, 400, and 500 μm the differences in the maximum temperature reached were significantly different from the other depths, except the immediately adjacent depths (Tukey post-hoc, *p *< 0.001). The delays were significantly different from all other delays except for the immediately adjacent depths (Tukey post-hoc, *p *< 0.001).

### Correlation between depth temperature and pain intensity

**Figure 9:**
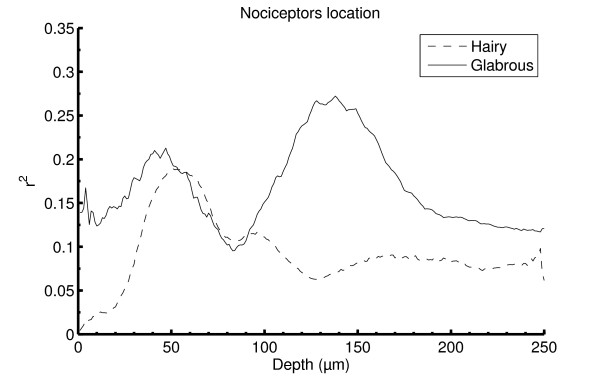
**The figure illustrates a linear regression analysis between the reported perception and the temperature profiles at varying depths**. Due to high fluctuations of the raw psychophysical data (VAS) a lowpass filter was applied for removing measuring artifacts (moving average filter, 5 points span inducing no time lag). For hairy skin the highest correlation was found at approximately 50 μm and for the glabrous skin the highest correlation was found at approximately 130 μm.

In hairy skin the strongest correlation between VAS and subsurface temperature was found for the temperature profile at 50 μm and with only one distinct peak (Figure 9). For glabrous skin, two peaks in the correlation coefficients were found; one at 50 μm but the strongest correlation was detected at 130 μm. These correlations might indicate the most likely receptor depth in hairy and glabrous skin.

## Discussion

A finite element 2D model for the cutaneous temperature distribution after infrared CO_2 _laser stimulation has been developed and validated. The modeled surface temperature distributions correlated with the measured surface skin temperatures and pain intensities following infrared CO_2 _laser stimulation in 16 healthy subjects. The model together with the experimental results indicated that the heat sensitive nociceptors are located more than two times deeper in glabrous skin (130 μm) than in hairy skin (50 μm).

### Model validation

When the modeled laser beam has a symmetrical axis, 2D axial modeling can be used to reduce the computational burden [11].

Different thermal properties of epidermis and dermis have been reported in the literature [12,16-19]. Hence, the thermal conductivity of epidermis varies from 0.17 W/(m·K) to 0.266 W/(m·K) and for the dermis between 0.29 and 0.58 W/(m·K). Hence, the thermal conductivity in dermis has been reported to be between 1.1 and 3.4 times greater than in epidermis. In the present study the values reported by Wilson and Spence [17], i.e. thermal conductivities of 0.21 W/(m·K) for epidermis, and 0.58 W/(m·K) for dermis were applied, based on the literature review made by Wilson and Spence [17] and the fact that several references state values close to 0.21 W/(m·K) for epidermis and 0.58 W/(m·K) for dermis [12,16-19].

A number of assumptions and limitations were needed when developing the model. The epidermal ridge and skin surface were modeled as being parallel, which is not correct, the epidermal ridge follows a certain rippled pattern and, therefore, the epidermal thickness is not constant but varies a few μm. Moreover, the epidermis and dermis were modeled as being completely homogenous tissues, which is a simplification, because the skin also contains other structures such as hair follicles, sweat glands and ducts. Neglecting the heat transfer due to radiation and convection was obviously a simplification but since only few blood vessels are present in the upper skin, convection only transfers a limited amount of the infrared heat energy [17]. It is typically assumed that for CO_2 _laser-tissue interactions, the main contributor to the heat transfer is conduction [17]. Furthermore, the lower boundary of the model was fixed at a constant temperature, meaning the model assumes that the heat energy is removed through the lower boundary, i.e. through the lower dermis. In the lower dermis larger blood vessels are present, which will transport the excess heat energy away and, thus, this assumption realistically models how the heat energy is removed from the stimulation site. This approach has previously been used by others with satisfying results [14]. However, despite these assumptions, the finite element model correlated well with the measured temperature at the skin surface. Finally, the absorption coefficient was based on Brugmans et al. [5] and a water content of the entire epidermis of 60%. However, the water content of the stratum corneum is lower than the rest of the living cells in epidermis. Since the absorption is proportional to the water content of the tissue [5], the stratum corneum will have a lower absorption than the rest of epidermis. Hence, in glabrous skin where the epidermal thickness is larger, mainly due to thicker stratum corneum, the laser energy will be absorbed slightly deeper than in hairy skin which might lead to a small error in the modeling of the laser absorption. However, correction of this would require measuring the thickness of the stratum corneum which is not feasible with the applied ultra sound scanner.

Although strong correlation was found between the maximum temperature of the FEM and experimental observations it is clear that there is less strong correlation between the model and the experimental data during the cooling phase (Figure 2). The cause of the slower predicted skin cooling in the FEM could be due to the fact that convection and radiation of heat were ignored in the model as discussed above.

The spatial analysis revealed that the temperature increase more than 0.5 cm from the beam center was identical for both types of 0.6 J stimulations (5 W, 0.12 s and 1 W, 0.6 s) (Figure 4). This occurs since the thermal energy is mainly transported into the skin and not spreading radially so the temperature change far away from the beam center will be similar when equal stimulation energy is applied.

### Physiological significance

The present results indicate that the perception latencies (defined as the reaction time from stimulus onset until the subject pressed a button) in glabrous skin was significantly slower than in hairy skin. A maximum difference of 0.8 s (Table 2, 5 W stimulation) between the two skin types was detected. One possible explanation is the distance to the spinal cord, the stimulations sites in the glabrous skin were placed more distally than those in the hairy skin. However, the conduction distance alone could not account for the 0.8 s difference in latencies between glabrous and hairy skin, which at most will be 0.2 s (10 cm difference in location and a conduction velocity of 0.5 m/s, assuming activation of the slowest conducting C fibers, conduction time will be 0.2 s). It has to be a combination of the thermal properties, different transduction mechanisms, including different receptor and nerve populations, and the skin locations.

**Figure 10:**
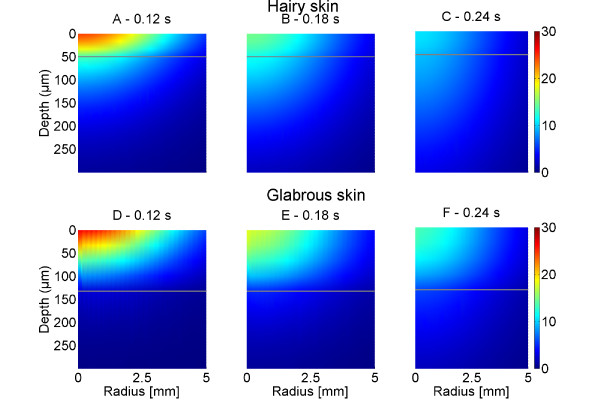
**The figure illustrates how the temperature is dispersed in hairy and glabrous skin following a 0.6 J/cm**^**2 **^**(5 W, 0.12 s) stimulation as a result of finite element modeling**. The plots represent the time at stimulus offset (A, D - 0.12 s), 1.5 × stimulus duration (B, E - 0.18 s) 2 × stimulus duration (C, F - 0.24 s). The horizontal grey line represents the epidermal ridge (50 μm in hairy skin and 130 μm in glabrous skin).

Only the 5 W power setting was used for estimating nociceptors depth, since many 1 W stimulations did not evoke any sensations. Treede et al. [3] reported that only the presence of type II AMHs (Aδ mechano-heat nociceptors) differs between hairy and glabrous skin. Evidently, CMH (C mechano-heat nociceptors) and type I AMH were present in both types of skin suggesting that any difference in activation of these fibers must reflect different localization in the skin and different thermal properties of the two types of skin. The present results indicated that equal stimulation in glabrous skin caused longer reaction times and less intense pain than in hairy skin, which could be caused by the different thermal properties of the skin types or the possible activation of type II AMHs when stimulating hairy skin. Assuming an intradermal initial temperature of 34°C and the threshold for activation of Aδ nociceptors is 46°C [3] this would correspond to a temperature increase of 12°C. The model indicates that an increase of 12°C can only occur at depths shallower than 100 μm. This is also demonstrated in Figure 10, which indicates the spatial subsurface heat distribution. Similar results have been obtained with an analytical modeling technique [14]. Both the current study and previous work [14] suggest that when using CO_2 _lasers more stimulation energy will be required to activate deeper located nociceptors.

Some subjects did report a pricking sensation (first pain) when stimulated on hairy skin, but no pricking sensations were reported when stimulating glabrous skin. Iannetti et al. [20] reported pricking pain sensations (reflecting activation of type II AMH fibers) from both hairy and glabrous skin using a laser with higher penetration than the CO_2 _laser which is in contradiction to Treede et al. [3,9], who only observed pricking pain in hairy skin. Iannetti et al. [20] suggested different epidermal thicknesses between the two skin types to account for different perceptions. Especially the stratum corneum in glabrous skin is often more than twice as thick as in hairy skin, and therefore, heat sensitive nociceptors must be located substantially deeper in the glabrous skin [20]. The present results provide further evidence for the location of nociceptors to depend on the skin type and hence epidermal thickness, but not to an extent that can explain the differences in perception latencies found in this study and reported by Treede et al. [3]. Therefore, the current study cannot reject the findings by Treede et al. [3]. Hence, in both hairy and glabrous skin the highest correlation to the pain intensity reports was found at the approximately same depth as the epidermal ridge (~ 50 μm in hairy skin and ~ 130 μm in glabrous skin), indicating the nociceptors to be placed at these depths. However, a second distinct peak for glabrous skin at approximately 50 μm is seen in Figure 9. This peak shows lower correlation than the peak at 130 μm. The reason for this peak could be due to a small subpopulation of fibers at this depth. Tillman et al. [6] found the mean depth of heat sensitive C fiber nociceptors to be 201 μm, which is well within the dermis, according to the present ultrasound measurements. Since neither of the subjects in the present study reported any pricking pain when stimulating the glabrous skin, this is in accordance with the results reported by Treede et al. [3,9]. To estimate the depth of Aδ fibers in the glabrous skin, a laser emitting light at shorter wavelengths, and thus, penetrating deeper, similar to the one used by Iannetti et al. [20], could be used. The maximum subsurface temperature has significantly larger delay in glabrous skin than in hairy skin, which indicates that a higher proportion of the transduction delay might be related to different thermal properties. The difference is most noticeable in the high power setting (5 W). For the low power setting (1 W), slower heating of the skin is induced so the difference in thermal conductivity of the dermis and epidermis plays a lesser role in the transmission of the heat energy. This finding is in accordance with the results of Tillman et al. [21] who evaluated how the thermal properties of the skin would affect the discharge of C fiber nociceptors (CMHs). This study concluded that due to the thermal inertia of the skin, the activation threshold temperature (at the skin surface) would increase with increasing stimulus ramp, this has been the debate of several publications [22-24]. Furthermore, the study by Tillman et al. [21] also concluded that a higher stimulus ramp would produce a higher peak discharge of nociceptors (CMHs) due to the phasic properties of C fiber nociceptors. Comparing this to the present study the highest pain evoked was during the high power setting/fast ramp (5 W), even though the stimulation energy was lower than the highest energy tested (1 W, 0.8 s - 0.8 J/cm^2^) which may support an effect of the stimulus ramp. Increasing the rate of the temperature change will reduce the pain ratings at the peak stimulus temperature [22], furthermore, the longer peak temperature duration, the higher pain ratings will be reported [22]. Both phenomenons can be explained based on the current study; first, when using a high rate of temperature change, the peak temperature is reached before the heat energy, absorbed at the skin surface, has diffused into the skin layers where the nociceptors are found. Second, when the peak temperature is maintained for a longer period the heat energy is allowed time to diffuse into the skin, causing higher nociceptor activation and resulting higher pain ratings. Pertovaara [23] found that the response latencies were decreased with higher stimulus ramp which is accordance with the data presented in this study.

As discussed above, the 5 W power setting will give the best indication of how large a fraction of the latency was due to different thermal properties due to the higher temperature gradient. Comparing the latency at 100 μm between glabrous and hairy skin, a latency difference of 0.04 s between the skin types was seen and increasing with depth. This means that approximately one third of the 0.15 s latency difference reported by Treede et al. [3] could be due to different thermal properties of the skin.

### Future applications of the model

The analytical expression for the heat source, Q, could be replaced with any equation, expressing the spatial profile and absorption of the laser light in the tissue. If using a more complex method of modeling the laser-tissue interactions, (for instance a Monte Carlo simulation), the result from this can easily be incorporated as the heat source in the present FEM which might render a more realistic temperature distribution in the skin.

## Conclusions

In the current study a 2D axial heat transfer model was developed to model the spatial heat distribution in human glabrous and hairy skin after irradiation by a CO_2 _laser. The geometry of the model was based on high resolution ultrasound scans. Based on experimental trials the model was validated. By combining the developed model and subjective pain ratings from 16 subjects it was possible to estimate the depth at which the heat-sensitive nociceptors are located in both hairy and glabrous skin. It was found that the heat-sensitive nociceptors in glabrous skin were located more than two times deeper than in hairy skin.

The developed model provides new possibilities of modeling the laser-skin interaction, including the possibility of modeling high penetration lasers.

## Competing interests

The authors declare that they have no competing interests.

## Authors' contributions

KSF carried out the FEM development, the experimental trials, analyzed the data, and drafted the manuscript. OKA participated in the design of the study, in the data analysis, and revised the manuscript. LAN participated in the design of the study, and revised the manuscript. CDM participated in the FEM development, the design of the study, in the data analysis, and revised the manuscript. All authors read and approved the final manuscript.
